# Supplementary material for: Passive motion of the lower extremities in sedated and ventilated patients in the ICU – a systematic review of early effects and replicability of Interventions
Source: PLoS One. 2022 May 12;17(5):e0267255. doi: 10.1371/journal.pone.0267255 (PMC9098053; doi:10.1371/journal.pone.0267255)
Supplement: S1 Checklist — PRISMA Checklist items in the manuscript. (PDF) [file pone.0267255.s001.pdf]

PRISMA 2020 Checklist

Section and Topic	Item #	Checklist item	Location where item is reported
**TITLE**			
Title	1	Identify the report as a systematic review.<br>"Passive motion of the lower extremities in sedated and ventilated patients in the ICU – a systematic review of early effects and replicability of interventions."	p. 1
**ABSTRACT**			
Abstract	2	See the PRISMA 2020 for Abstracts checklist.	p. 3-4
**INTRODUCTION**			
Rationale	3	Describe the rationale for the review in the context of existing knowledge.<br>"Due to the unstable condition at the beginning of their disease, critically ill patients often require mechanical ventilation and analgosedation. In consequence, immobility occurs, which leads to muscle degradation up to 30% already within ten days of inactivity, which is primarily reflected in a reduced size of muscle fibers [1, 2]. In addition, the patients often suffer from systemic inflammation because of shock, trauma, sepsis or due to the critical illness itself and it is known that in this context pro-inflammatory cytokines increase the degradation of muscle proteins [3].<br>Both immobility and inflammation are among the most frequently mentioned risk factors that, in combination with the existing severe illness, favor the "intensive care unit-acquired weakness" (ICU-AW) [4]. This complication affects up to 67% of patients who have been ventilated for more than ten days and 80% of critically ill patients [4-6]. ICU-AW clinically manifests as symmetrical flaccid paresis of the extremities and not only prolongs the stay in the intensive care unit (ICU), but is also associated with increased morbidity and mortality [7]. Furthermore, ICU-AW, although reversible in principle, often leads to disability that lasts until after the acute hospital [3, 5]. Especially the strength of the lower extremities often remains permanently limited, which negatively affects the quality of life after surviving a severe illness due to the reduced ability to walk [8, 9].<br>It is reported that the concept of early mobilization (active and passive, in and out of bed) leads to decreased incidence of ICU-AW, shorter delirium duration, more ventilator-free days, improved muscle strength, decreased muscle atrophy and length of hospital stay, a better functional outcome at hospital discharge and increased discharged-to-home rate for patients with a critical illness [10-15].<br>Due to cardiac, haemodynamic or pulmonary instability and the resulting need for ventilation and analgosedation, it is usually not yet possible for patients to actively cooperate and getting mobilized in a chair during the first few days in the ICU. However, early mobilization of critically ill patients in the ICU is recommended within 48 to 72 hours after the start of mechanical ventilation, whenever possible [1, 16].<br>Passive movements and bed cycling are recommended in sedated patients with a RASS (Richmond Agitation Sedation Scale) ≥ -3 [17] and as early as possible in the course of treatment [18]. Despite the widespread use of the recommendations for passive movements and bed cycling and their reported safety and feasibility [19-22], these interventions lack a firm evidence-base concerning the effectiveness of passive motion treatment methods in bedridden patients [23-25], notwithstanding this patient population is at greatest risk of developing ICU-AW [24].<br>One study result indicates continuous passive motion (CPM) to significantly reduce muscle fibre atrophy and protein loss, when it is compared to standard therapy [26]. Another study reports passive cycling to result in significantly improved muscle strength after the intervention [27]. However, whether the passive movements can prevent muscle tissue from atrophy remains unclear in both studies [26, 27].<br>Data from other studies suggest passive bed cycling to improve anti-inflammatory processes and the immune response in critically ill patients and thus could prevent muscle degradation and the development of ICU-AW [28].<br>Yet, what is unclear is whether passive early motion measures of the lower extremities used in sedated and ventilated ICU patients reduces muscle wasting, has positive effects on inflammation and the immune system and could, therefore, prevent the development of ICU-AW."	p. 5-6

Section and Topic	Item #	Checklist item	Location where item is reported
Objectives	4	Provide an explicit statement of the objective(s) or question(s) the review addresses.<br>"This systematic review aims to summarize the effects of passive motion of the lower extremities in sedated and ventilated patients in the ICU on musculature, inflammation, immune system and the development of ICU-AW. Furthermore, the aim was to evaluate the replicability of the used interventions and the methodology of the included studies for practical settings."	p. 7
**METHODS**			
Eligibility criteria	5	Specify the inclusion and exclusion criteria for the review and how studies were grouped for the syntheses.<br>"Studies were included in this review if they were 1) randomized controlled trials 2) in English or German language, 3) included mechanically and invasively ventilated and sedated critically ill patients ≥ 18 years, who require treatment in the ICU in an acute hospital, 4) evaluated the effect of passive motion of the lower extremities carried out in bed, either manually or through a therapy device, on musculature, inflammation, the immune system or the development of ICU-AW and 5) included a comparison group that received either no therapy, standard therapy or a different dosage of the intervention.<br>Passive motion is used as a generic term for all passive measures that counteract the negative consequences of immobility in patients requiring intensive care. The measure is carried out manually or by means of a therapy device, is performed within the bed and results in movement without the active cooperation of the patient. As long as the passive measures take place in the ICU, they are counted as "early" at any time - i.e. even after 72h after admission to the ICU. Sedated patients were defined as deeply sedated to non-awakable (RASS -4 to -5, Ramsay Score > 4). Standard therapy was defined as respiratory therapy or nursing measures and positioning.<br>Studies with children, with animals or those written in another language were excluded. In addition, studies with passive early motion in combination with other early rehabilitation measures, such as active early motion, electrotherapy or mobilization out of bed, were excluded."	p. 8-9
Information sources	6	Specify all databases, registers, websites, organisations, reference lists and other sources searched or consulted to identify studies. Specify the date when each source was last searched or consulted.<br>"The electronic literature search was conducted by a professional librarian at the University of Zurich (SK), in the databases Medline, Embase, Cochrane Library, CINAHL and PEDro up to 6th May 2020. The complete search strategy is stated in S2 Appendix. To check whether further relevant RCT studies were published after May 2020, an additional search of the databases up to 20th February 2022 was performed."	p. 7 and S2 Appendix
Search strategy	7	Present the full search strategies for all databases, registers and websites, including any filters and limits used.<br>"The terms population, intervention and study design were used to develop the search strategy (Table 1). The search strategy contained MeSH terms (critical illness, intensive care units, bed rest / immobilization, humans / adult, deep (conscious) sedation, respiration, artificial, motion therapy, continuous passive, exercise therapy, lower extremity, physical therapy specialty / modalities, rehabilitation, movement, bicycling) and free text words (critically ill, sedat*, mechanical ventilat*, early passive exerc* / cycl* / mobile* / «range of motion» / therap* / treat* / train*, leg / limb, bed exercise, recumbent / in bed, kinetic therapy). In addition, the search strategy was restricted to randomized controlled trials."	p. 7-8 and S2 Appendix
Selection process	8	Specify the methods used to decide whether a study met the inclusion criteria of the review, including how many reviewers screened each record and each report retrieved, whether they worked independently, and if applicable, details of automation tools used in the process.<br>"After conducting the literature search, duplicates were screened out by SK. The screening of the studies was carried out in several steps by two independent reviewers (NH, RV) using the defined inclusion and exclusion criteria. In case no agreement could be reached between these two reviewers, RK acted as an independent referee.<br>In a first step, NH and RV performed a title and abstract screening to remove obviously irrelevant references. The refences were therefore marked as "clearly include", "maybe include" or "clearly exclude". The second step involved full-text screening with the references that remained. Finally, the third step involved a hand search by RV reviewing the reference lists of the included studies. In addition, RV contacted authors to ask for missing information that would allow inclusion of the studies."	p. 9-10
Data collection process	9	Specify the methods used to collect data from reports, including how many reviewers collected data from each report, whether they worked independently, any processes for obtaining or confirming data from study investigators, and if applicable, details of automation tools used in the	p. 10

Section and Topic	Item #	Checklist item	Location where item is reported
		process.<br>“Following the Cochrane checklist, the first author extracted data on 1) reference with publication year, 2) study design, 3) population, 4) setting, 5) intervention, 6) control group, 7) outcome variables and 8) results [35]. To describe the population age, gender, duration of ventilation at the start of the intervention, sedation depth, severity of illness and reason for admission to the ICU were extracted. The presentation of the measures implemented in the intervention and control group is based on the reporting of Frequency, Intensity, Time, and Type (FITT) intervention components [36]. The measurement methods and the timing of the measurements are presented for the relevant target variables. In addition, the group differences, the significance level and the effect size are described for the results.<br>In case the corresponding data were not presented in an included study, RV contacted the authors concerned in order to be able to include the data in this review.”	
Data items	10a	List and define all outcomes for which data were sought. Specify whether all results that were compatible with each outcome domain in each study were sought (e.g. for all measures, time points, analyses), and if not, the methods used to decide which results to collect.<br>“Following the Cochrane checklist, the first author extracted data on 1) reference with publication year, 2) study design, 3) population, 4) setting, 5) intervention, 6) control group, 7) outcome variables and 8) results [35]. To describe the population age, gender, duration of ventilation at the start of the intervention, sedation depth, severity of illness and reason for admission to the ICU were extracted. The presentation of the measures implemented in the intervention and control group is based on the reporting of Frequency, Intensity, Time, and Type (FITT) intervention components [36]. The measurement methods and the timing of the measurements are presented for the relevant target variables. In addition, the group differences, the significance level and the effect size are described for the results.<br>In case the corresponding data were not presented in an included study, RV contacted the authors concerned in order to be able to include the data in this review.”<br>“The description of the intervention was assessed by two independent investigators (RV and AM). The items of the TIDieR Checklist were rated as either “sufficient” (+) or as “not / not adequately” reported (-).<br>In the event of disagreements between AM and RV, RK was called in as referee.”	p. 10
	10b	List and define all other variables for which data were sought (e.g. participant and intervention characteristics, funding sources). Describe any assumptions made about any missing or unclear information.<br>“Following the Cochrane checklist, the first author extracted data on 1) reference with publication year, 2) study design, 3) population, 4) setting, 5) intervention, 6) control group, 7) outcome variables and 8) results [35]. To describe the population age, gender, duration of ventilation at the start of the intervention, sedation depth, severity of illness and reason for admission to the ICU were extracted. The presentation of the measures implemented in the intervention and control group is based on the reporting of Frequency, Intensity, Time, and Type (FITT) intervention components [36]. The measurement methods and the timing of the measurements are presented for the relevant target variables. In addition, the group differences, the significance level and the effect size are described for the results.<br>In case the corresponding data were not presented in an included study, RV contacted the authors concerned in order to be able to include the data in this review.”	p. 10
Study risk of bias assessment	11	Specify the methods used to assess risk of bias in the included studies, including details of the tool(s) used, how many reviewers assessed each study and whether they worked independently, and if applicable, details of automation tools used in the process.<br>The methodological quality of the randomized controlled trials was assessed by two independent reviewers (AM and RV) using the Downs and Black checklist [37]. It was agreed that 0 points (for "no" or "unclear") or 1 point (for "yes") would be awarded for each item. For item 4, one point is only awarded in case all FITT criteria were described [36]. When no information was described for item 19 (assessment of compliance with the intervention), this was rated as "yes" in the ICU setting and 1 point was awarded. Items 9 and 26 were scored as "yes" and 1 point when the loss-to-follow-up rate was reported and was < 15%. Finally, for Item 27, 1 point was awarded when a sample size calculation was performed and 0 points when this item was missing. These adjustments lead to a total possible score of 27 points.<br>In the event of disagreements between AM and RV, RK was called in as referee.	p. 10-11

PRISMA 2020 Checklist

Section and Topic	Item #	Checklist item	Location where item is reported
Effect measures	12	Specify for each outcome the effect measure(s) (e.g. risk ratio, mean difference) used in the synthesis or presentation of results.<br>“To show the effect of the interventions, the standardized mean difference (SMD), the so-called effect size, was calculated for the randomized controlled trials that reported the mean and standard deviation of the relevant outcome variables. The effect size was determined by calculating Cohen's d, whereby a d between 0.2 and 0.5 indicates a small effect, between 0.5 and 0.8 a medium effect, and a d greater than 0.8 indicates a strong effect [38].<br>In order to show the agreement of the reviewers for the assessment of the risk of bias with the Downs and Black checklist, the Cohen's Kappa coefficient was calculated [39]. A kappa value below 0.20 is considered a weak agreement, between 0.21 and 0.40 a slight agreement, between 0.41 and 0.60 a moderate agreement, between 0.61 and 0.80 a good agreement, and greater than 0.81 a very good agreement [40].”	p. 11
Synthesis methods	13a	Describe the processes used to decide which studies were eligible for each synthesis (e.g. tabulating the study intervention characteristics and comparing against the planned groups for each synthesis (item #5)).<br>A meta-analysis was intended to be provided when sufficient data would be available. On P11<br>&<br>No quantitative syntheses were performed due to the heterogeneity of the outcome measures and insufficient methodological quality of included studies. P21.	p. 11<br><br>p. 21
	13b	Describe any methods required to prepare the data for presentation or synthesis, such as handling of missing summary statistics, or data conversions.	NA
	13c	Describe any methods used to tabulate or visually display results of individual studies and syntheses.	NA
	13d	Describe any methods used to synthesize results and provide a rationale for the choice(s). If meta-analysis was performed, describe the model(s), method(s) to identify the presence and extent of statistical heterogeneity, and software package(s) used.	NA
	13e	Describe any methods used to explore possible causes of heterogeneity among study results (e.g. subgroup analysis, meta-regression).	NA
	13f	Describe any sensitivity analyses conducted to assess robustness of the synthesized results.	NA
Reporting bias assessment	14	Describe any methods used to assess risk of bias due to missing results in a synthesis (arising from reporting biases).	NA
Certainty assessment	15	Describe any methods used to assess certainty (or confidence) in the body of evidence for an outcome.	NA
**RESULTS**			
Study selection	16a	Describe the results of the search and selection process, from the number of records identified in the search to the number of studies included in the review, ideally using a flow diagram.<br>The literature search (search strategy S2 Appendix) resulted in 2810 hits. After removing the duplicates, the number of references was reduced to 1817. After the additional search, no studies could be identified<br>The Flow Chart in Figure 1 show the process of study selection based on the predefined inclusion and exclusion criteria. After the title and abstract screening, 1740 studies could be excluded. The remaining 77 studies were read in full text. This full text screening led to the exclusion of a further 72 studies due to inappropriate population, intervention or control group, different language or because they were not clinical trials. No further study could be included by hand-searching of the references of the selected manuscripts. However, contacting three authors resulted in further study inclusion. Finally, five studies were integrated into the qualitative synthesis.<br>A search of the databases between May 2020 and February 2021 did not result in additional references.<br>Figure 1. Flow Chart. Presentation of the study selection process [33]	p. 12 and Figure 1
	16b	Cite studies that might appear to meet the inclusion criteria, but which were excluded, and explain why they were excluded.	NR

Section and Topic	Item #	Checklist item	Location where item is reported
Study characteristics	17	Cite each included study and present its characteristics.<br>“The characteristics of the five included studies [24, 26, 41-43] are presented in Table 2. All studies were conducted in intensive care units, three in Brazil, one in the United Kingdom, and one in France.<br>The total of 87 patients (19 women and 49 men, 19 patients’ sex unclear) were on average 56.4 years old. The number of days with ventilation before the start of the study was only stated in two studies and ranged from a median of 4 days [24] to 6.44 ± 0.333 days in the intervention group and 4.9 ± 2.80 days in the control group [42]. All patients in the included studies were deeply sedated (RASS -4 or -5, Ramsay score 6), comatose or relaxed. Severity of the disease was stated in three studies with the APACHE II score and showed in average 18.6 points and, with 24%, therefore a low average risk of dying in hospital [26, 42, 43]. In another study the SAPS II score was used and a value of 57.5 ± 24 suggests a 50-75% in-hospital mortality [24]. The reason for admission to the ICU was missing in two studies [41, 43]. In the other studies, respiratory failure or sepsis is most frequently mentioned [24, 26, 42].<br>In the included studies, three different passive early motion interventions were described for ventilated and sedated patients in the ICU. Passive exercise was performed by a therapist as a stand-alone intervention [24] or combined with the application of a cuff to restrict blood flow [41]. Passive bed cycling has been described in three studies [24, 42, 43] but each performed with a different dosage. Finally, in one study passive motion was performed by a CPM machine [26]. Apart from one study, in which the ankle joint was moved in isolation [26], the remaining studies involved movement of the entire lower extremity (hip, knee and ankle joint movement).<br>The outcome categories of musculature, inflammatory factors, immune system and development of ICU-AW were measured with different parameters for the same target variables in the included studies. Only muscle thickness was measured in two studies [41, 44] and also with the same measurement method. In the other studies the target variables and how these were methodologically measured differed between the reports. The target variables were measured before and then directly after the intervention [24], 1 h after the intervention [42], 7 days after the start of the intervention [26, 43] or as soon as the patients were no longer sedated and could respond to requests [41].<br>Effects of passive interventions with group differences, p-value, and calculated effect sizes are reported in Table 2.”	p. 12-16 and table 2
Risk of bias in studies	18	Present assessments of risk of bias for each included study.<br>“The included studies achieved an average score of 19.8, with a median of 22 points on the Downs and Black Checklist [37]. An overview is presented in Table 4. Three studies scored the highest with 22 points [24, 41, 43] and one scored the lowest with 13 points [26].<br>The total per item reported ranged from 0 to 5 points with a median of 4 points. In all studies, full scores were obtained on twelve items (2, 3, 5-7, 10, 14, 16, 17, 19, 22, 23). In contrast, item 12 ("Were those subjects who were prepared to participate representative of the entire population from which they were recruited?" [37] and item 25 ("Was there adequate adjustment for confounding in the analyses from which the main findings were drawn?" [37]) was rated with 0 points in all studies.”	p. 17-19 and table 4
Results of individual studies	19	For all outcomes, present, for each study: (a) summary statistics for each group (where appropriate) and (b) an effect estimate and its precision (e.g. confidence/credible interval), ideally using structured tables or plots.	p. 14-16 in table 2
Results of syntheses	20a	For each synthesis, briefly summarise the characteristics and risk of bias among contributing studies.	NA
	20b	Present results of all statistical syntheses conducted. If meta-analysis was done, present for each the summary estimate and its precision (e.g. confidence/credible interval) and measures of statistical heterogeneity. If comparing groups, describe the direction of the effect.	NA
	20c	Present results of all investigations of possible causes of heterogeneity among study results.	NA
	20d	Present results of all sensitivity analyses conducted to assess the robustness of the synthesized results.	NA
Reporting biases	21	Present assessments of risk of bias due to missing results (arising from reporting biases) for each synthesis assessed.	NA
Certainty of evidence	22	Present assessments of certainty (or confidence) in the body of evidence for each outcome assessed.	NA

PRISMA 2020 Checklist

Section and Topic	Item #	Checklist item	Location where item is reported
**DISCUSSION**			
Discussion	23a	Provide a general interpretation of the results in the context of other evidence.<br>"The results of this systematic review show a slight tendency of benefits related to passive motion in sedated and ventilated patients in the ICU observed in the muscle structure, in the microcirculation and in the inflammation factors and the immune system. Conversely, the efficacy for early and intensive passive movement in immobilized ICU patients could not yet be determined, as the summarized evidence was retrieved of studies with small samples. Moreover, these studies are partly replicable and their effect sizes are difficult to derive from these studies.<br>Then again, three of the included studies showed slight positive benefits of passive motion on muscle fibers, protein loss and muscle thickness and circumference, when compared to standard therapy or no intervention, without being able to completely prevent muscle loss [26, 41, 43].<br>Griffiths et al. measured significant preservation of muscle fibers and prevention of protein loss after passive movement in the ankle joint by a CPM machine in the more severely ill patients [26]. It can be considered beneficial that an effect of the intervention was evident, although only a small muscle group was passively moved and not the whole lower extremity as it was performed in the other studies [24, 41-43]. Though it must be noted that the generalizability of this study may be questioned due to the small number of patients (n = 5) and because of the deficiencies in the methodological quality. Moreover, the dosage of the intervention in this study contained a total of 9h per day, which is hardly feasible in today's daily routine in the ICU. Comparable interventions with relaxed patients were not performed since the latter publication, as relaxants for a longer time are hardly necessary nowadays with the newer ventilation technology and the existence of short-acting medications [26]. The results of significant preservation of muscle fibers are in line with the findings from the study of Llano-Diez et al. which demonstrated the effect of a high dosage of passive movement with a CPM machine [27]. In this report deeply sedated ventilated patients with brain injury of whom an extremity was passively moved by a CPM machine during 9 ± 1 days for 10 hours per day (4x 2.5 h) were observed. The study reported that the specific force of single muscle fibers in the intervention leg was 35% higher compared to the leg without passive movement. However, because this study lacked randomization the results should be interpreted with care.<br><br>The benefits observed on muscle structure through changes in microcirculation is described by the study of Barbalho et al., where muscle circumference and muscle thickness could be maintained through passive manual exercise with a blood pressure cuff [41]. This is the first study to investigate the application of a cuff to restrict blood flow in patients requiring intensive care. However, this intervention is already successfully used in musculoskeletal rehabilitation. A systematic review with blood flow restriction in addition with low-impact training showed a moderate effect on muscle strength gain compared to training alone in patients with knee osteoarthritis, ligament injuries or myositis and in older patients who are prone to sarcopenia [47]. Positive effects on the microcirculation can therefore indirectly protect the muscles, because temporary reduction in blood flow leads to a counter-reaction of the body and, in combination with exercise, even if it is low-dose, can support the maintenance or build-up of muscle mass [41]. On the other hand, some results suggested that passive motion applied manually or by a bed cycle led to higher microcirculation, and is therefore supposed to improve blood circulation in the muscles and could reduce muscle loss [24, 41]. Whether a higher microcirculation can actually positively affect muscles needs further investigation.<br><br>Another benefit of passive motion could be seen in one of the included studies on inflammation and immune system. A single 20-minute session of passive exercise on the bed cycle showed a beneficial impact on reducing inflammation, as shown by significant reduction of nitrosative stress, measured by NO production in stimulated (C+) and non-stimulated (C-) monocytes in the blood. Furthermore, there seemed to be a positive influence on the immune system by significantly reducing the inflammatory effective TNF-α and increasing anti-inflammatory cytokine IL-10 [42]. The positive tendency of passive motion on nitrosative stress and inflammation has also been demonstrated in other studies. A study with rats described that unilateral passive mechanical loading resulted in a reduction of oxidative stress and attenuated the loss of muscle mass and force-generation capacity and could therefore show beneficial effects on muscle size and function [48]. Amidei et al. investigated the effect of passive movement through a CPM machine on cytokines in a quasi-experimental study [28]. 30 patients with mechanical ventilation were passively moved for 20 min at a rate of 20 flexion/extension movements in the knee joint per minute within 72	p. 22-24

Section and Topic	Item #	Checklist item	Location where item is reported
		hours. The patients were not all deeply sedated (GCS 3-13 at baseline), which means that active movements can distort the results. The intervention showed a significant reduction in IL-6, which indicates an anti-inflammatory effect of the intervention. IL-10 showed no significant change between baseline measurement and 60 min after the intervention. However, it is noteworthy that the ratio between IL-6 and IL-10 was also calculated in the study. This ratio could be clinically relevant, as IL-6 is a pro-inflammatory cytokine and IL-10 is an anti-inflammatory cytokine. The study showed that this ratio improved with the intervention, which certainly required a change in IL-10 concentration, but the IL-10 concentration may only be significantly reduced with a time delay."	
	23b	Discuss any limitations of the evidence included in the review.<br>"A typical problem of research with critically ill ICU patients are the many confounding factors that can distort the effect of the interventions.<br>First, the influence of medications is an important confounding factor. For example, the administration of corticosteroids and muscle relaxants increase the risk of developing ICU-AW [60, 61]. Corticosteroids also have an anti-inflammatory effect and support the immune system. In the case of increased signs of infection, patients in the ICU receive targeted anti-inflammatory drug therapy, whereby the administration of vasoconstrictive catecholamines can lead to reduced microcirculation in the case of haemodynamic instability [62]. Thus, within the included studies of this systematic review, drugs could have had a major impact on outcome parameters.<br>Secondly, patient nutrition must be mentioned as another confounding factor. As nutrition can have an important impact on limiting the muscle loss associated with critical illness, only with an optimal nutritional status under the prevailing conditions can its influence, for example on muscle loss, be kept as small as possible in order to demonstrate the isolated effect of an intervention [58]. The results of an ongoing study by Zhou et al. could provide information about the interaction of exercise and nutrition [63].<br>Thirdly, critically ill patients in intensive care often suffer from numerous comorbidities or may have installations which inhibit movements, that can have an influence on muscle loss, for example neurological diagnosis, sarcopenia, the need for extracorporeal membrane oxygenation etc. [7, 61, 64]. These factors can distort the effect of passive early motion. Clear and comparable inclusion and exclusion criteria and an accurate description of the included patients are helpful to compare the patient populations of different studies.<br><br>Apart from the confounding factors, the present work revealed that a clear definition of early mobilization and a core set of outcome variables for early mobilisation in the ICU are missing [65]. A defined set of outcome variables would allow comparability of studies. The core set should include target variables at the cellular and structural level as well as at the functional level. Furthermore, it must be possible to capture both the acute effects of the interventions and the long-term effects. On structural level it seems reasonable to measure muscle thickness with ultrasound, as this outcome measure is shown to be reliable and valid in the population of critically ill patients on the ICU [66, 67]. On the other hand, performing a muscle biopsy provides important information about the muscle fibers, but carries a higher risk of complications due to its invasiveness and can only be performed by specialists. Furthermore, on functional level the MRC and MRC sum score can deliver information about muscle strength and the clinical diagnosis of an ICU-AW. As though a blood analysis can provide interesting information on cellular level by measuring oxidative and nitrosative stress and cytokines, these outcome measures must be performed in a specialized laboratory and are therefore more complicated to involve in everyday working life. If it is possible, especially the ratio of IL-6 and IL-10 seems to be clinically relevant [28].<br><br>In addition, studies with larger populations, longer intervention periods or higher doses, as well as the start of early mobilization within 48-72 h in the intensive care unit are lacking.<br>Furthermore, it would be informative to investigate whether additional passive early mobilisation also prevents muscle breakdown in ventilated patients with mild sedation and has a positive effect on inflammation, the immune system and the development of ICU-AW. This is because even in this population, only a few active interventions are often possible due to reduced exercise capacity.<br>It is important to investigate the effectiveness of individual interventions in high-quality studies with large populations and to describe the intervention completely in order to be able to implement positive study results in everyday clinical practice. In this way, the effective interventions can be applied in a targeted manner in intensive care units to those patients who can actually benefit from them. Especially in	p. 26-28

Section and Topic	Item #	Checklist item	Location where item is reported
		connection with Covid-19, effective passive early mobilisation measures could be of great importance, as the affected patients usually have to be sedated for a long time and often also have to be relaxed and show a high incidence of ICU-AW after re-awakening [68]. The results of our review show, however, that many reports lack a clear description of the interventions which hinders their full replication in a clinical setting."	
	23c	Discuss any limitations of the review processes used.<br>"Our systematic review has some limitations that should be mentioned. First, the included studies evaluated the effects of the interventions with different methodological measures, which prohibited to conduct a meta-analysis.<br>Secondly, it must be mentioned that the calculated effect sizes can be affected by biases of individual studies. Thirdly, a language bias must be assumed in the conduct of the present systematic review due to the exclusion of studies in a language other than German and English. And finally, publication bias cannot be ruled out."	p. 28-29
	23d	Discuss implications of the results for practice, policy, and future research.<br>"Future studies should possibly record both the acute and the long-term effects of the interventions. Furthermore, studies with larger populations, longer intervention periods or higher dosages, rigorous methodological quality as well as the start of early mobilisation within 48-72 h in the intensive care unit are required. These studies should preferably adhere to guidelines for reporting, e.g. the Tidier checklist, since that would allow the full replication of successful interventions in clinical settings."	p. 29
**OTHER INFORMATION**			
Registration and protocol	24a	Provide registration information for the review, including register name and registration number, or state that the review was not registered.<br>"…the review was not registered."	p. 2
	24b	Indicate where the review protocol can be accessed, or state that a protocol was not prepared.<br>"A protocol was conducted in advance of the study."	p. 2
	24c	Describe and explain any amendments to information provided at registration or in the protocol.	NA
Support	25	Describe sources of financial or non-financial support for the review, and the role of the funders or sponsors in the review.	NA
Competing interests	26	Declare any competing interests of review authors.<br>"The authors have declared that no competing interests exist."	p. 2
Availability of data, code and other materials	27	Report which of the following are publicly available and where they can be found: template data collection forms; data extracted from included studies; data used for all analyses; analytic code; any other materials used in the review.<br>"All relevant data are within the paper."	p. 2
