# Supplementary material for: Passive motion of the lower extremities in sedated and ventilated patients in the ICU – a systematic review of early effects and replicability of Interventions
Source: PLoS One. 2022 May 12;17(5):e0267255. doi: 10.1371/journal.pone.0267255 (PMC9098053; doi:10.1371/journal.pone.0267255)
Supplement: S2 Checklist — PRISMA for Abstracts Checklist items in the manuscript. (PDF) [file pone.0267255.s002.pdf]

PRISMA 2020 for Abstracts Checklist

Section and Topic	Item #	Checklist item	Reported (Yes/No)
**TITLE**			
Title	1	Identify the report as a systematic review.<br>"The aim of the study is to systematically review the currently available randomized controlled trials…"	Yes
**BACKGROUND**			
Objectives	2	Provide an explicit statement of the main objective(s) or question(s) the review addresses.<br>"Early mobilization, which includes active / passive motion in bed along with mobilization out of bed, is recommended to prevent the development of intensive care unit acquired-weakness (ICU-AW) for patients with critical illness on the intensive care unit. To date, the impact of passive motion of the lower extremities in sedated and ventilated patients remains unclear.<br>The aim of the study is to systematically review and summarize the currently available randomized controlled trials in English or German language on the impact of passive motion of the lower extremities in sedated and ventilated patients ≥ 18 years in the intensive care unit on musculature, inflammation and immune system and the development of intensive care unit-acquired weakness and to evaluate the replicability of interventions and the methodological quality of included studies."	Yes
**METHODS**			
Eligibility criteria	3	Specify the inclusion and exclusion criteria for the review.<br>"The aim of the study is to systematically review and summarize the currently available randomized controlled trials in English or German language on the impact of passive motion of the lower extremities in sedated and ventilated patients ≥ 18 years in the intensive care unit on musculature, inflammation and immune system and the development of intensive care unit-acquired weakness and to evaluate the replicability of interventions and the methodological quality of included studies."	Yes
Information sources	4	Specify the information sources (e.g. databases, registers) used to identify studies and the date when each was last searched.<br>"A systematic literature search was performed up to 20th February 2022 in the databases Medline, Embase, Cochrane Library, CINAHL and PEDro."	Yes
Risk of bias	5	Specify the methods used to assess risk of bias in the included studies.<br>"… the methodological quality (Downs and Black checklist) were assessed."	Yes
Synthesis of results	6	Specify the methods used to present and synthesise results.<br>"The description of the intervention (TIDieR checklist) and the methodological quality (Downs and Black checklist) were assessed."	Yes
**RESULTS**			
Included studies	7	Give the total number of included studies and participants and summarise relevant characteristics of studies.	Yes

Section and Topic	Item #	Checklist item	Reported (Yes/No)
		"Five studies were included in the qualitative syntheses. On average, the studies were rated with 6.8 out of 12 points according to the TIDieR checklist. For the methodological quality an average of 19.8 out of 27 points on the Downs and Black checklist was reported. The results of included studies indicated that muscle loss may be reduced by passive manual movement, passive cycling and passive motion on a continuous passive motion-unit. In addition, positive effects were reported on the reduction of nitrosative stress and the immune response. The impact on the development of ICU-AW remains unclear."	
Synthesis of results	8	Present results for main outcomes, preferably indicating the number of included studies and participants for each. If meta-analysis was done, report the summary estimate and confidence/credible interval. If comparing groups, indicate the direction of the effect (i.e. which group is favoured).<br>"Five studies were included in the qualitative syntheses. On average, the studies were rated with 6.8 out of 12 points according to the TIDieR checklist. For the methodological quality an average of 19.8 out of 27 points on the Downs and Black checklist was reported. The results of included studies indicated that muscle loss may be reduced by passive manual movement, passive cycling and passive motion on a continuous passive motion-unit. In addition, positive effects were reported on the reduction of nitrosative stress and the immune response. The impact on the development of ICU-AW remains unclear."	Yes
**DISCUSSION**			
Limitations of evidence	9	Provide a brief summary of the limitations of the evidence included in the review (e.g. study risk of bias, inconsistency and imprecision).<br>"Future randomized controlled trials should use larger samples, use complete intervention description, use a comparable set of outcome measures, use rigorous methodology and examine the effect of passive motion on the development of ICU-AW."	Yes
Interpretation	10	Provide a general interpretation of the results and important implications.<br>"In conclusion, passive movement show a slight tendency for beneficial changes on cellular level in sedated and ventilated patients in the ICU within the first days of admission, which may indicate a reduction of muscle wasting and could prevent the development of ICU-AW."	Yes
**OTHER**			
Funding	11	Specify the primary source of funding for the review.	No
Registration	12	Provide the register name and registration number.	No

From: Page MJ, McKenzie JE, Bossuyt PM, Boutron I, Hoffmann TC, Mulrow CD, et al. The PRISMA 2020 statement: an updated guideline for reporting systematic reviews. BMJ 2021;372:n71. doi: 10.1136/bmj.n71
