# Supplementary material for: Passive motion of the lower extremities in sedated and ventilated patients in the ICU – a systematic review of early effects and replicability of Interventions
Source: PLoS One. 2022 May 12;17(5):e0267255. doi: 10.1371/journal.pone.0267255 (PMC9098053; doi:10.1371/journal.pone.0267255)
Supplement: S1 File — Summary of the complete search strategy. (PDF) [file pone.0267255.s003.pdf]

S2 Appendix. Complete search strategy.

a) Search strategy Medline

Ovid® Wolters Kluwer

My Account | Ask a Librarian | Support & Training | University of Zürich | Help | Feedback | Logoff

Search **Journals** **Books** **Multimedia** **My Workspace** **Links**

▼ **Search History** (5) View Saved

# ▲	Searches	Results	Type	Actions	Annotations
2	exp exercise therapy/ or exp rehabilitation/ or Movement/ or exp Bicycling/ or exp Lower Extremity/ or (physical therap* or physiotherap* or motion therap* or readaptation* or rehabilitation* or readaption* or readjustment* or exerc* or kinesiotherap* or kinesitherap* or bicycling or cycling or cycle ergometer or move* or train* or leg* or mobili* or kinetic therap*).ti,ab. or (lower adj2 (extremit* or limb*)).ti,ab.	2154599	Advanced	Display Results | More	
3	(passiv* or (early adj3 (rehab* or mobili* or motion*))).ti,ab.	134010	Advanced	Display Results | More	
4	(randomized controlled trial or controlled clinical trial).pt. or (randomized or placebo or randomly).ti,ab. or trial.ti. or exp clinical trial/	1394540	Advanced	Display Results | More	
5	1 and 2 and 3 and 4	378	Advanced	Display Results | More	

Expand

b) Search strategy Embase

Embase®

Embase Session Results (6 May 2020)

No.	Query	Results
#5	#1 AND #2 AND #3 AND #4	865
#4	'crossover procedure':de OR 'double-blind procedure':de OR 'randomized controlled trial':de OR 'single-blind procedure':de OR random*:de,ab,ti OR factorial*:de,ab,ti OR crossover*:de,ab,ti OR ((cross NEXT/1 over*):de,ab,ti) OR placebo*:de,ab,ti OR ((doubl* NEAR/1 blind*):de,ab,ti) OR ((singl* NEAR/1 blind*):de,ab,ti) OR assign*:de,ab,ti OR allocat*:de,ab,ti OR volunteer*:de,ab,ti	2548739
#3	passiv*:ti,ab OR ((early NEAR/3 (rehab* OR mobili* OR motion*)):ti,ab)	159211
#2	'physiotherapy'/exp OR 'rehabilitation'/exp OR 'leg movement'/exp OR 'kinesiotherapy'/exp OR 'cycling'/exp OR 'lower limb'/exp OR 'physical therap*':ti,ab OR physiotherap*:ti,ab OR 'motion therap*':ti,ab OR readaptation*:ti,ab OR rehabilitation*:ti,ab OR readaption*:ti,ab OR readjustment*:ti,ab OR exerc*:ti,ab OR kinesiotherap*:ti,ab OR kinesitherap*:ti,ab OR bicycling:ti,ab OR cycling:ti,ab OR 'cycle ergometer':ti,ab OR move*:ti,ab OR train*:ti,ab OR leg*:ti,ab OR mobili*:ti,ab OR 'kinetic therap*':ti,ab OR ((lower NEAR/2 (extremit* OR limb*)):ti,ab)	2941239
#1	'intensive care'/de OR 'artificial ventilation'/exp OR 'intensive care unit'/exp OR 'critical illness'/exp OR 'bed rest'/exp OR 'immobility'/exp OR (((intensive OR critical*) NEAR/3 (care* OR therap* OR treat* OR ill*)):ti,ab) OR icu:ti,ab OR icuaw:ti,ab OR sedat*:ti,ab OR ventilat*:ti,ab OR recumbent*:ti,ab OR 'in bed':ti,ab	812949

ELSEVIER

© 2020 Elsevier Life Sciences IP Limited except certain content provided by third parties.
Embase is a trade mark of Elsevier Life Sciences IP Limited. RELX Group and the RE symbol are trade marks of RELX Group plc, used under license.

c) Search strategy Cochrane Library

Cochrane Library

Access provided by: **UZH Hauptbibliothek / Zentralbibliothek Zürich**

Advanced Search

Search manager

Save this search | View saved searches | ? Search help

Print

#1	(((intensive OR critical*) NEAR/3 (care* OR therap* OR treat* OR ill*) OR ICU OR ICUAW OR sedat* OR ventilat*) OR (recumbent* OR "in bed")):ti,ab,kw		Limits	78465
#2	(("physical therap*" OR physiotherap* OR "motion therap*" OR readaptation* OR rehabilitation* OR readaption* OR readjustment* OR exerc* OR kinesiotherap* OR kinesitherap* OR bicycling OR Cycling OR "cycle ergometer" OR move* OR train* OR leg* OR mobili* OR "kinetic therap*") OR ((lower NEAR/2 (extremit* OR limb*)))):ti,ab,kw		Limits	246963
#3	(passiv* OR (early NEAR/3 (rehab* OR mobili* OR Motion*))):ti,ab,kw		Limits	10952
#4	#1 AND #2 AND #3		Limits	690
#5	Type a search term or use the S or	S MeSH	Limits	N/A

Clear all

Highlight orphan lines

Save this search | View saved searches | ? Search help

Print

d) Search strategy CINAHL

EBSCOhost

Wednesday, May 06, 2020 6:29:05 AM

#	Query	Limiters/Expanders	Last Run Via	Results
S5	S1 AND S2 AND S3 AND S4	Expanders - Apply equivalent subjects Search modes - Find all my search terms	Interface - EBSCOhost Research Databases Search Screen - Advanced Search Database - CINAHL with Full Text	862
S4	TX (double-blind OR random* OR control*) OR TI trial	Expanders - Apply equivalent subjects Search modes - Find all my search terms	Interface - EBSCOhost Research Databases Search Screen - Advanced Search Database - CINAHL with Full Text	2,694,327
S3	TI (passiv* OR (early N3 (rehab* OR mobili* OR motion*))) OR AB (passiv* OR (early N3 (rehab* OR mobili* OR motion*)))	Expanders - Apply equivalent subjects Search modes - Find all my search terms	Interface - EBSCOhost Research Databases Search Screen - Advanced Search Database - CINAHL with Full Text	23,812
S2	((MH "Physical Therapy+") OR (MH "Rehabilitation+") OR (MH "Movement+") OR (MH "Cycling") OR (MH "Lower Extremity+")) OR TI ((physical therap* OR physiotherap* OR motion therap* OR readaptation* OR rehabilitation* OR readaption* OR readjustment* OR exerc* OR kinesiotherap* OR kinesitherap* OR bicycling OR cycling OR cycle ergometer OR move* OR train* OR leg* OR mobili*	Expanders - Apply equivalent subjects Search modes - Find all my search terms	Interface - EBSCOhost Research Databases Search Screen - Advanced Search Database - CINAHL with Full Text	962,234

	OR kinetic therap*) OR ((lower N2 (extremit* OR limb*)))) OR AB ((physical therap* OR physiotherap* OR motion therap* OR readaptation* OR rehabilitation* OR readaption* OR readjustment* OR exerc* OR kinesiotherap* OR kinesitherap* OR bicycling OR cycling OR cycle ergometer OR move* OR train* OR leg* OR mobili* OR kinetic therap*) OR ((lower N2 (extremit* OR limb*))))			
S1	((MH "Critical Care+") OR (MH "Respiration, Artificial+") OR (MH "Intensive Care Units+") OR (MH "Critical Illness") OR (MH "Bed Rest") OR (MH "Immobilization")) OR TI (((intensive OR critical*) N3 (care* OR therap* OR treat* OR ill*) OR ICU OR ICUAW OR sedat* OR ventilat*) OR (recumbent* OR in bed)) OR AB (((intensive OR critical*) N3 (care* OR therap* OR treat* OR ill*) OR ICU OR ICUAW OR sedat* OR ventilat*) OR (recumbent* OR in bed))	Expanders - Apply equivalent subjects<br>Search modes - Find all my search terms	Interface - EBSCOhost Research Databases<br>Search Screen - Advanced Search<br>Database - CINAHL with Full Text	235,440

e) Search strategy PEDro

Pedro – in 4 Schritten

Title & abstract: intensive care

Title only: early mobili*

Method: clinical trial

Title & abstract: critical* ill*

Title only: early mobili*

Method: clinical trial

Title & abstract: intensive care

Title only: passiv*

Method: clinical trial

Title & abstract: critical* ill*

Title only: passiv*

Method: clinical trial
