# Supplementary material for: Passive motion of the lower extremities in sedated and ventilated patients in the ICU – a systematic review of early effects and replicability of Interventions
Source: PLoS One. 2022 May 12;17(5):e0267255. doi: 10.1371/journal.pone.0267255 (PMC9098053; doi:10.1371/journal.pone.0267255)
Supplement: Attachment — Submitted filename: Response to Reviewers.docx [file pone.0267255.s004.docx]

**Reviewer's report**

**Title:** Passive motion of the lower extremities in sedated and ventilated patients in the ICU – a systematic review of early effects and replicability of interventions

**Version:** 2 **Date:** 16 February 2022

**Reviewer:** #1

**Reviewer's report:**

Dr Vollenweider and colleagues report the results of a systematic review of RCTs investigating the effects of early passive motion of the lower limbs in sedated and mechanically ventilated critically ill patients, on muscle mass preservation and function.

The main findings are 1) that such RCT are scarce, as the authors could find only 5, 2) that those RCTs used different outcome measures, and 3) that they were all small sized, as overall the systematic review involved only 87 patients. The great heterogeneity in methods used for passive motion and in outcome measures across studies precluded the conduct of a metaanalysis.

The authors' conclusion is that the effects of early passive motion in the targeted population are possibly beneficial on a cellular level but that larger size, well conducted randomized studies are needed to 1) confirm the effects of passive motion of the lower limbs, and 2) investigate whether such effects could translate into less muscle wasting during the ICU stay and better muscle function and physical autonomy in the mid- or long-term.

The systematic review is appropriately conducted. The manuscript is well-written and concise. Tables, figures, and Appendices are informative.

I have a few minor comments or suggestions:

-L212, “The Flow Chart in Figure 1 showed the process”. I would write “show” instead of “showed”.

***Our answer:***

***We thank the reviewer for this helpful comment. We have followed the advice and changed “showed” to “show”. (New L214)***

-L317,”… and therefore indicate the importance of early…”. I would temper this assertion because of very small sized studies that were not replicated. I also would add the notion that an estimate of the effect size is very difficult to derive from those studies.

***Our answer:***

***We thank the reviewer for this helpful comment. We have followed the advice and edited the manuscript to read:***

***New L320-324 Conversely, the efficacy for early and intensive passive movement in immobilized ICU patients could not yet be determined, as the summarized evidence was retrieved from studies with small samples. Moreover, these studies are only partly replicable, and the effect sizes are difficult to derive from these studies.***

***Then again,..***

L-356, “…Whether a higher microcirculation can actually positively effect muscles needs…”. It seems it should be “affect”.

***Our answer:***

***We thank the reviewer for this helpful comment. We have followed the advice and changed “effect” to “affect”. (new L361)***

**Reviewer's report**

**Title:** Passive motion of the lower extremities in sedated and ventilated patients in the ICU – a systematic review of early effects and replicability of interventions

**Version:** 2 **Date:**16 February 2022

**Reviewer:** ACGastaldi

**Reviewer's report:**

It is an elegant review properly performed according to systematic review guidelines.

This topic is important but the 5 selected studies, one of these with only 5 patients, makes the results and conclusion weak.

Because of this, I do not agree with the conclusion in the abstract:

'This review indicates early passive motion to benefit on cellular and structural level in sedated and ventilated patients.'

It needs to be changed according to the paper conclusion, that has more caution:

'In conclusion, passive movement show a slight tendency for beneficial changes on cellular level in sedated and ventilated patients in the ICU within the first days of admission, which may indicate a reduction of muscle wasting and could prevent the development of ICU-AW.'

***Our answer:***

***We thank the reviewer for this helpful comment and adopted the proposed phrase. The abstract was edited accordingly and now reads:***

***In conclusion, passive movements show a slight tendency for beneficial changes on cellular level in sedated and ventilated patients in the ICU within the first days of admission, which may indicate a reduction of muscle wasting and could prevent the development of ICU-AW. (L53-55)***
